# Structure-Function Analysis of PPP1R3D, a Protein Phosphatase 1 Targeting Subunit, Reveals a Binding Motif for 14-3-3 Proteins which Regulates its Glycogenic Properties

**DOI:** 10.1371/journal.pone.0131476

**Published:** 2015-06-26

**Authors:** Carla Rubio-Villena, Pascual Sanz, Maria Adelaida Garcia-Gimeno

**Affiliations:** Instituto de Biomedicina de Valencia, CSIC, and Centro de Investigación en Red de Enfermedades Raras (CIBERER), Jaime Roig 11, Valencia, Spain; Louisiana State University Health Sciences Center, UNITED STATES

## Abstract

Protein phosphatase 1 (PP1) is one of the major protein phosphatases in eukaryotic cells. It plays a key role in regulating glycogen synthesis, by dephosphorylating crucial enzymes involved in glycogen homeostasis such as glycogen synthase (GS) and glycogen phosphorylase (GP). To play this role, PP1 binds to specific glycogen targeting subunits that, on one hand recognize the substrates to be dephosphorylated and on the other hand recruit PP1 to glycogen particles. In this work we have analyzed the functionality of the different protein binding domains of one of these glycogen targeting subunits, namely PPP1R3D (R6) and studied how binding properties of different domains affect its glycogenic properties. We have found that the PP1 binding domain of R6 comprises a conserved RVXF motif (R_102_VRF) located at the N-terminus of the protein. We have also identified a region located at the C-terminus of R6 (W_267_DNND) that is involved in binding to the PP1 glycogenic substrates. Our results indicate that although binding to PP1 and glycogenic substrates are independent processes, impairment of any of them results in lack of glycogenic activity of R6. In addition, we have characterized a novel site of regulation in R6 that is involved in binding to 14-3-3 proteins (RARS_74_LP). We present evidence indicating that when binding of R6 to 14-3-3 proteins is prevented, R6 displays hyper-glycogenic activity although is rapidly degraded by the lysosomal pathway. These results define binding to 14-3-3 proteins as an additional pathway in the control of the glycogenic properties of R6.

## Introduction

The control of glycogen homeostasis occurs via an exquisite coordination of events. These events comprises from the regulation of glucose intake to the control of glycogen synthesis and breakdown, amongst others. The key enzymes involved in glycogen metabolism are the glycogen synthase (GS) and glycogen phosphorylase (GP). The dephosphorylation of these enzymes by the protein phosphatase 1 (PP1) results in the stimulation of glycogen synthesis by activating GS, and the prevention of glycogen breakdown by inactivating GP, which leads to the net accumulation of the polysaccharide [1]. However, these PP1 glycogenic substrates establish only weak interactions with the phosphatase catalytic subunit (PP1c), thus the process requires the mediation of PP1 regulatory subunits to allow an efficient dephosphorylation ([2], [3]). In this context, it has been described until now seven glycogen targeting subunits [PPP1R3A (GM), PPP1R3B (GL), PPP1R3C (R5/PTG), PPP1R3D (R6), PPP1R3E (R3E), PPP1R3F (R3F) and PPP1R3G (R3G); [1], [3]] that serve as scaffold proteins. These glycogen targeting subunits not only provide additional docking sites for PP1 glycogenic substrates but also recruit the phosphatase to the glycogen particle, where the concentration of the substrates is higher. Therefore, to accomplish their function, the glycogen targeting subunits need to bind to the PP1c catalytic subunit, to the PP1 glycogenic substrates and also to the glycogen particle ([1], [2], [3]).

PP1c is one of the major protein phosphatase involved in many different processes in eukaryotic cells. The specificity for the substrates that is able to dephosphorylate is given by its binding to a particular regulatory subunit. At present, more than one hundred different PP1 regulatory subunits have been defined [4], and although they do not show any overall degree of homology, most of them share a common docking motif for PP1 binding, named the RVXF motif ([2], [3]). This motif is present in the glycogen targeting subunits described above [5], although its functionality has only been proven in GM (R_63_VSF) ([6], [7]), GL (R_62_VSF) ([6], [7]), R5/PTG (R_84_VVF) [8] and R3F (R_36_VLF) [9]. These glycogenic subunits also bind to the PP1 substrates (i.e., GS and GP) to allow their efficient dephosphorylation by the PP1 phosphatase. It was postulated that binding of glycogen targeting subunits to these substrates was mediated by a conserved sequence WXNXGNYX(L/I) [5]. However, at present, the functionality of this domain has only been demonstrated in the case of GM (W_219_SNNN, [10]) and R5/PTG (W_222_DSNR, [11]). Finally, these glycogenic subunits contain a carbohydrate binding module of the CBM21 type ([12], [13]) that allows their binding to the glycogen particle [5]. This property is crucial for the localization of the PP1 phosphatase to this specific subcellular compartment where the glycogenic substrates are present.

In this work, we have characterized the different binding domains of the glycogen targeting subunit PPP1R3D (R6) and have evaluated their functionality in regulating glycogen production. R6 is a glycogenic subunit of 33 kDa widely distributed in a variety of tissues, including liver, skeletal muscle, pancreas and brain ([14], [15]). In muscle cells R6 has a clear glycogenic activity, which is higher than GM but lower that R5/PTG [16]. We have recently described that the glycogenic activity of R6 is regulated by ubiquitination: R6 interacts with laforin, a dual specificity phosphatase involved in Lafora disease (a type of progressive myoclonus epilepsy), which targets R6 to malin, an E3-ubiquitin ligase also related to Lafora disease [17]. The action of the laforin-malin complex results in the monoubiquitination and also in the polyubiquitination (through K63-linked chains) of R6, which results in an impairment of the glycogenic activity of this glycogen targeting subunit and the degradation of R6 through the lysosomal pathway [17]. Recently, in a high-throughput screening, it was found that R6 potentially interacted with 14-3-3 proteins [18]. 14-3-3 family of proteins bind to Ser/Thr phosphorylated residues on target proteins producing a variety of different responses: for example, they can occlude a docking region of the target protein, affect to the subcellular localization or provoke a conformational change [19]. 14-3-3 proteins interact with their targets mainly via a consensus sequence RSXpSXP [19], although it has been recently found that other residues outside the canonical motif may be required to strength binding [20]. Here we show that R6 possesses a consensus motif for 14-3-3 protein binding, RARS_74_LP, not present in other glycogenic subunits such as R5/PTG or GL, and demonstrate that binding to 14-3-3 proteins affects the glycogenic properties of R6 and its rate of lysosomal degradation.

## Materials and Methods

### Plasmids

pFLAG-R6, pBTM-R6, pGADT7-R6, pACT2-laforin, pACT2-PP1α and pEYFP-R6 constructs were described previously ([17], [21], [22], [23]). pACT2-14-3-3ε plasmid was a generous gift from Dra. Lynne Yenush (IBMCP, UPV-CSIC, Valencia, Spain). Mutant constructs pEYFP-R6 S25A, S74A, RARA, RAHA, WDNAD and WANNA were obtained by site directed mutagenesis using Quick-Change Mutagenesis kit and the corresponding mutagenic oligonucleotides (see Table 1), according to the manufacturer’s protocol. Mutations were all confirmed by DNA sequencing. The corresponding ORFs were subcloned into yeast pBTM116 and mammalian pFLAG-6c vectors to allow their expression either in yeast or in mammalian cells.

**Table 1:** Primers used for site directed mutagenesis.

MUTATION	FORWARD PRIMER	REVERSE PRIMER
S25A	AAG CTC GGC CCC CGG AGC CTC GCA TGC CTG TCG GAC CTG GAC GGC	GCC GTC CAG GTC CGA CAG GCA TGC GAG GCT CCG GGG GCC GAG CTT
S74A	ATC ATC CTG CGG CGG GCC CGG GCA CTG CCC AGC TCC CCC GAG CGC	GCG CTC GGG GGA GCT GGG CAG TGC CCG GGC CCG CCG CAG GAT GAT
V103A-F105A (RARA)	TGC AGC CAG AAG CTC CGC GCC CGG GCC GCC GAC GCC CTG GGC TTG	CAA GCC CAG GGC GTC GGC GGC CCG GGC GCG GAG CTT CTG GCT GCA
V253A-F255A (RAHA)	GAG CTC GGC TCC CGC GCG CAC GCC GCG GTG CGC TAC CAA	TTG GTA GCG CAC CGC GGC GTG CGC GCG GGA GCC GAG CTC
N270A (WDNAD)	GCC GAG TAC TGG GAC AAC GCC GAC CAC AGA GAC TAC AGC	GCT GTA GTC TCT GTG GTC GGC GTT GTC CCA GTA CTC GGC
D268A-D271A (WANNA)	GCG GGT GCC GAG TAC TGG GCC AAC AAC GCC CAC AGA GAC TAC AGC	GCT GTA GTC TCT GTG GGC GTT GTT GGC CCA GTA CTC GGC ACC CGC

### Cell culture, transfection and treatments

Murine neuroblastoma Neuro-2a (N2a) and human embryonic kidney (Hek293) cells (from the Health Protection Agency Culture Collection, Salisbury, UK) were grown in Dulbecco’s modified Eagle’s medium (Lonza, Barcelona, Spain), supplemented with 100 units/ml penicillin, 100 μg/ml streptomycin, 2 mM glutamine and 10% of inactivated fetal bovine serum (Invitrogen, Madrid, Spain) in a humidified atmosphere at 37°C with 5% CO_2_. Cells were transfected with 1 μg of each plasmid using either X-treme GENE HP transfection reagent (Roche Diagnostics, Barcelona, Spain) or Lipofectamine 2000 (Invitrogen, Madrid, Spain), according to the manufacturer’s instructions. When indicated, 18 hours after transfection, cells were treated with MG132 (5 μM) or ammonium chloride (20 mM)/ leupeptin (100 μM) for 6 hours. Alternatively, cells were also treated with cycloheximide (300 μM) for the indicated times.

### Preparation of crude extracts and immunoblotting

Cell extracts were prepared using lysis buffer [25 mM Tris-HCl at pH 7.5, 15 mM EDTA at pH 8, 50 mM NaF, 0.6 M sucrose, 15 mM 2-mercaptoethanol, 15 mM Na_4_P_2_O_7_, 1 mM PMSF, and a complete Mini-EDTA free protease inhibitor mixture (Roche Diagnostics, Barcelona, Spain)]. Cells were lysed by repeated passage through 24Gx5/8” needle and centrifuged at 13,000xg 10 min. Thirty micrograms of total protein from the soluble fraction of cell lysates were analyzed by SDS-PAGE and Western blotting using appropriated antibodies: anti-FLAG, anti-HA and anti-actin (Sigma-Aldrich, Madrid, Spain); anti-tubulin, anti-LexA and anti-PP1α (Santa Cruz Biotechnology, Barcelona, Spain); anti-GS (rabbit monoclonal antibody against the C-term of muscular glycogen synthase) and anti-GP (mouse monoclonal against the muscular isoform of the glycogen phosphorylase) (Abcam, Cambridge Science Park, UK); anti-14-3-3ε (Abgent, San Diego, USA) and anti-GFP (ImmunoK, AMS Biotechnology LTD.). Secondary antibodies were from Santa Cruz Biotechnology (Barcelona, Spain). Immunoblots were analyzed by using ECLprime reagent (GE Healthcare, Barcelona, Spain) and chemiluminescence was detected using a Fujifilm LAS- 4000 Lite imager.

### Yeast two-hybrid analysis

Yeast THY-AP4 strain [*MATα ura3 leu2 trp1 lexA*::*lacZ lexA*::*HIS3 lexA*::*ADE2*] [24] was co-transformed with plasmids pBTM-R6 (encoding LexA-R6 wild type and the corresponding mutants) and pACT2 (GAD, empty plasmid), pACT2-PP1α (GAD-PP1), pACT2-laforin (GAD-laforin) or pACT2-14-3-3ε (GAD-14-3-3). Yeast transformants were grown in selective SC medium and β-galactosidase activity was assayed in permeabilized cells and expressed in Miller units as in [25]. Protein levels in crude extracts were analyzed by western blotting as in [17].

### Immunoprecipitation (GFP-Trap analysis)

Hek293 cells were transiently transfected with expression vectors coding for YFP, YFP-R6 wild type, YFP-R6 S25A, YFP-R6 S74A, YFP-R6 RARA, YFP-R6 RAHA, YFP-R6 WDNAD or YFP-R6 WANNA plasmids. After twenty-four hours of transfection, cells were scraped on ice with 1 mL ice cold PBS and transferred to a pre-cooled tube to spin the cells at 500xg for 3 min. After two PBS washes, pelleted cells were resuspended in 200 μL lysis buffer [25 mM Tris-HCl pH 7,5; 150 mM NaCl, 0,5 mM EDTA, 5% glycerol, 0,5% nonidet P-40 (NP-40), complete protease inhibitor cocktail (Roche Diagnostics, Barcelona, Spain), 1 mM PMSF, 5 mM NaF and 5 mM Na_4_P_2_O_7_]. Cell lysates were placed on ice for 30 min and centrifuged at 13,000×g for 15 min at 4°C. An aliquot of the supernatant was used to determine protein concentration (Bradford method). Two mg of the supernatant was incubated under rotation with 20 μL of pre-equilibrated GFP-Trap_A bead slurry (Chromotek, Germany) for 10 min at 4°C. After extensive washing, beads were resuspended with 100 μL of 2xSDS-sample buffer, eluted by heating 10 min at 95°C and centrifuged. 40 μL of eluted beads and thirty micrograms of total protein from the soluble fraction of cell lysates were analyzed by SDS-PAGE and Western blotting using appropriated antibodies.

### Multiple sequence alignment and homology model

Amino acid sequences of PPP1R3B (GL), PPP1R3C (R5) and PPP1R3D (R6) from *H*. *sapiens* were obtained from Uniprot database (accession numbers Q86XI6, Q9UQK1 and O95685, respectively) and aligned using the Clustal Omega program (http://www.ebi.ac.uk/Tools/msa/clustalo/). To adjust the alignment we used secondary structure prediction (GOR4 program, [26]). Homology model was generated by SWISS-MODEL Workspace ([27], [28]) based on template of PPP1R3B (GL) (pdb accession code: 2EEF; Tomizawa et al., unpublished data). Quality of protein model was estimated by QMEAN4 [29] and Ramachandran plot evaluation by RAMPAGE program [30]. Images were generated using PyMol (DeLano Scientific LLC, USA). PhosphoSite was used for consensus phosphorylation site detection (www.phosphosite.org).

### Glycogen determination

Neuro-2a cells were transfected with the indicated plasmids. Forty-eight hours after transfection, cells were scraped on ice into 30% KOH and then heated at 100°C for 15 min. Glycogen was measured as described previously [31] and expressed as μg of glucose per mg of protein.

### Immunofluorescence and confocal microscopy

Neuro-2a cells transfected with the indicated plasmids were grown on 12-well plates containing coverslips. Cells were fixed with 4% paraformaldehyde in phosphate-buffered saline (PBS) for 10 min. Then, they were permeabilized with 0.2% Triton X-100 in PBS for 15 min, washed three times with PBS and blocked one hour with 10% fetal bovine serum, 5% nonfat dried milk, 0,5% bovine serum albumin, and 0.1% Triton X-100 in PBS. Cells were then incubated overnight at 4°C with 1/200 dilution of anti-glycogen antibody (a generous gift from Dr. Otto Baba; Tokyo Medical and Dental University, Japan). Samples were washed three times with PBS and incubated with a 1/500 dilution of anti-mouse IgM antibodies coupled to Alexa-Fluor 633. Then, cells were washed three times with PBS and mounted on slices using Aqua-Poly/Mount coverslipping medium (Polysciences, Inc. Eppelheim, Germany). For R6 and glycogen subcellular localization, images were acquired with a Leica TCS SL confocal microscope (Leica, Wetzlar, Germany) using a PL APO 63× oil 1.4 N.A. immersion objective. Images were treated with the ImageJ 1.43c software (Wayne Rasband, National Institutes of Health, Bethesda, MD).

### Statistical data analysis

Data are expressed as means with standard deviation. Statistical significance of differences between the groups was evaluated by a paired Student’s t-test with two-tailed distribution. The significance has been considered at *p<0.05, **p<0.01, ***p<0.001, as indicated in each case.

## Results

### Characterization and structural modelling of R6 protein domains

**Fig 1:**
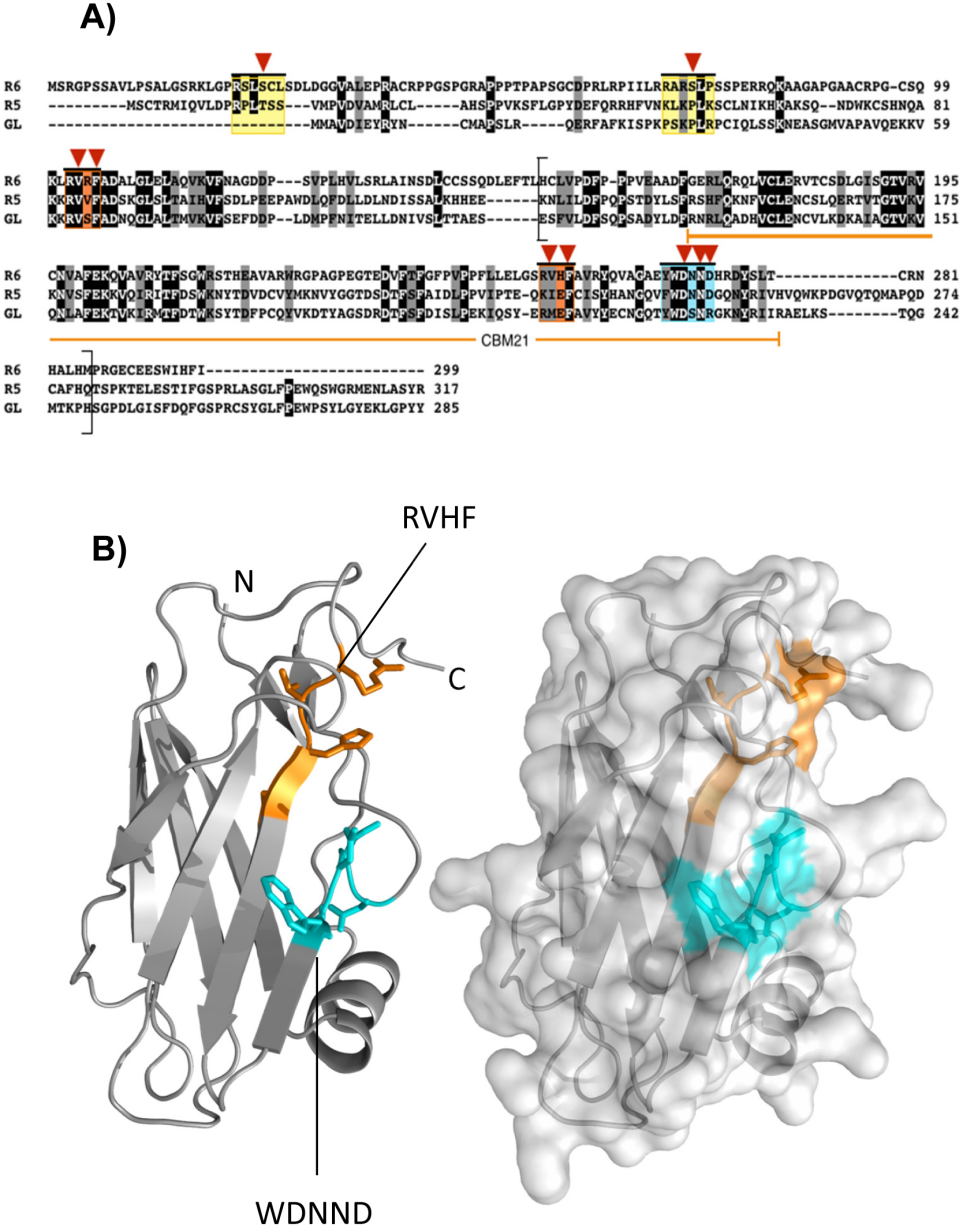
Characterization and structural modelling of R6 domains. (A) Multiple sequence alignment of PPP1R3D (R6), PPP1R3C (R5) and PPP1R3B (GL) proteins sequences from *H*. *sapiens* (Uniprot entries: O95685, Q9UQK1 and Q86XI6, respectively) was performed using the Clustal Omega program (see Materials and Methods). Invariance and conservation are highlighted by black and gray shadowing respectively. Amino acid sequences for the binding to 14-3-3 proteins, PP1 catalytic subunit and PP1 glycogenic substrates are enclosed in yellow, orange and cyan boxes respectively. Brackets indicate the modeled sequence of R6 shown in panel B. The CBM21 domain of R6 (according to Uniprot) is underlined in orange. Red triangles point at the mutated residues obtained in this study. (B) Homology model of the CBM21 domain of R6 was based on the template of GL (pdb entry: 2EEF). The amino acids corresponding to the PP1 glycogenic substrate binding domain (W_267_DNND) and to the putative RVXF (R_252_VHF) are shown in sticks and colored in cyan or orange, respectively (left panel). The N- and C-terminus of the model is also indicated. A representation of the surface of the R6 homology model to show the exposed amino acids to the solvent is presented in the right panel. Images were generated using PyMol (DeLano Scientific LLC, USA).

A multiple sequence protein alignment between PPP1R3B (GL), PPP1R3C (R5/PTG) and PPP1R3D (R6) of *H*. *sapiens* (Fig 1A) allowed the definition of different characteristic protein domains in R6. As PP1 glycogen-targeting subunits, all these proteins possess protein motifs for binding to both the PP1 catalytic subunit (PP1c) and the glycogenic PP1 substrates [i.e. glycogen synthase (GS), glycogen phosphorylase (GP)]. The named-RVXF- motif is the primary PP1-docking motif present in about 70% of all PP1 interacting proteins ([2], [3]). Two putative RVXF sequences can be found in R6, R_102_VRF and R_252_VHF. However, only the R_102_VRF sequence was conserved in the three mammalian subunits tested (Fig 1A, first orange box), being the second putative R_252_VHF motif only present in R6 (Fig 1A, second orange box at the C-terminus). It has been described that the binding of glycogenic targeting subunits to glycogen-related protein substrates occurs via a single highly conserved WDNNE/D motif ([10], [11]). This motif was located at the C-terminus of R6 (W_267_DNND) (Fig 1A, cyan box). In addition, glycogenic targeting subunits contain a carbohydrate binding module of the CBM21 type ([12], [13]) that allows binding of these subunits to glycogen. In the case of R6 this domain spans from residue 169 to 278 (underlined region in Fig 1A).

To estimate the relative position of these motifs in the overall structure of the protein we generated an homology model using as a template the NMR structure of the GL protein (pdb accession code: 2EEF). To validate the model we used secondary structure prediction from the protein sequence and analyzed the Ramachandran plot, which had good statistics (88.4% in the favored region). The model spans from residue 153 to 286 in R6 (Fig 1A, region within brackets), covering the entire CBM21 domain and containing the second putative R_252_VHF motif and the W_267_DNND motif involved in binding to PP1 substrates (Fig 1B). The modelled region shows a typical β-barrel structure, with seven β-strands arranged into two major β-sheets. One of these β-sheets consists of one parallel and one antiparallel β-strand pair, whereas the second sheet is a four-stranded antiparallel β-sheet. The model also shows the presence of an α-helix that is located on one side of the β-barrel structure (Fig 1B). It is interesting to note that in this model the W_267_DNND motif is clearly exposed to the solvent, what would allow the interaction of R6 with PP1 substrates. On the contrary, the putative R_252_VHF motif seems to be partially buried in the model, what suggests poor PP1 binding properties for this motif (Fig 1B).

### Assessing the functionality of the putative RVXF motifs of R6 in PP1c binding

**Fig 2:**
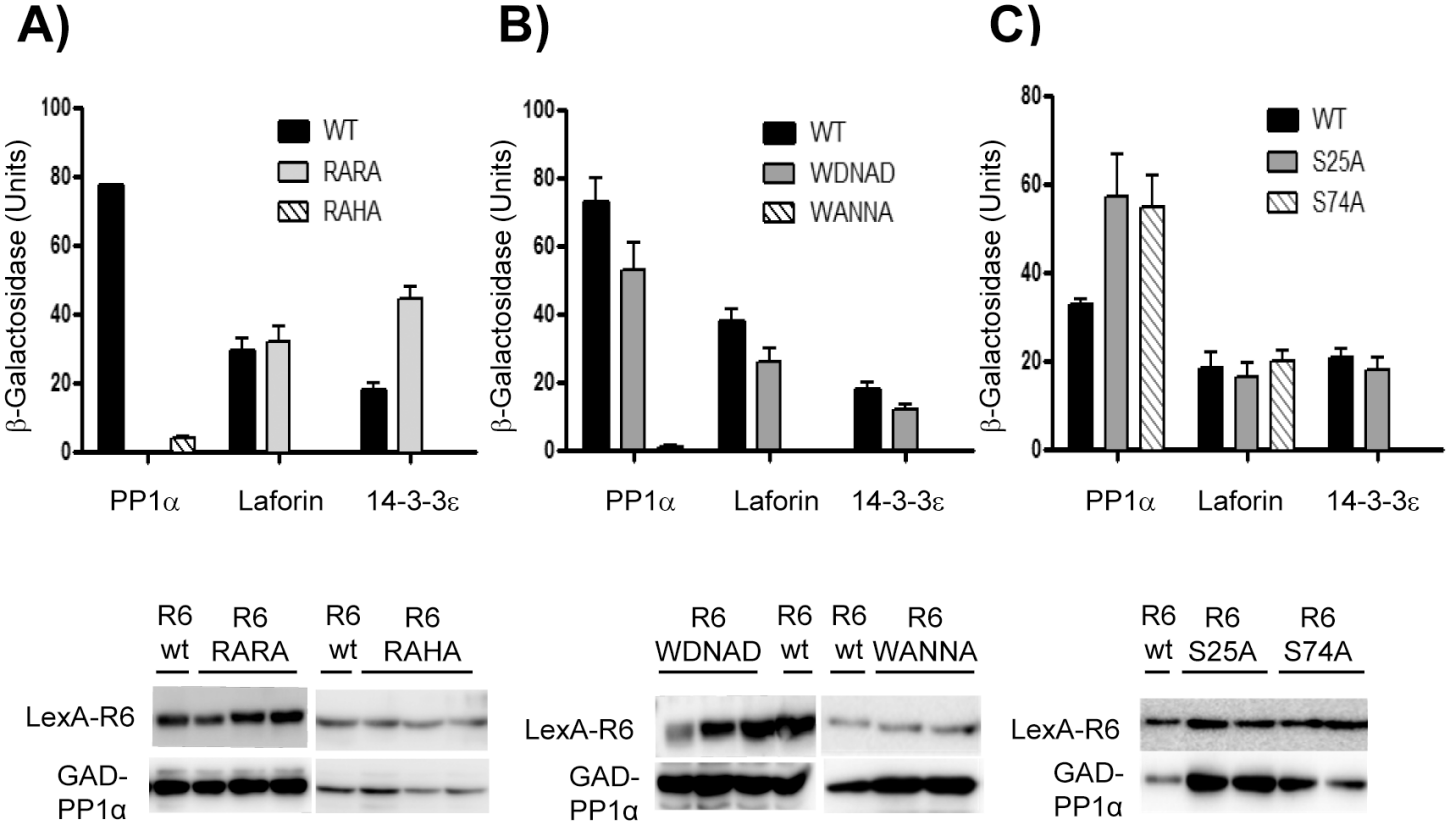
Analysis of the interacting properties of different domains of R6 by yeast two-hybrid analyses. Upper panels: yeast THY-AP4 strain was transformed with plasmids pBTM-R6 wt (LexA-R6), pBTM-R6 RARA and pBTM-R6 RAHA (A), pBTM-R6 WDNAD, and pBTM-WANNA (B) or pBTM-R6 S25A and pBTM-R6 S74A (C) and with pACT2 (GAD), pACT2-PP1α (GAD-PP1α), pACT2-laforin (GAD-laforin) or pACT2-14-3-3ε (GAD-14-3-3ε). Protein interaction was estimated by measuring the β-galactosidase activity. Values correspond to means from at least 6 different transformants (bars indicate standard deviation). Lower panels: protein expression in yeast transformants was analyzed by Western blotting using anti-HA antibodies (for the GAD-fusions) and anti-LexA (for the LexA-fusions) in several transformants from each condition. A representative western blot of some of these transformants is shown.

**Fig 3:**
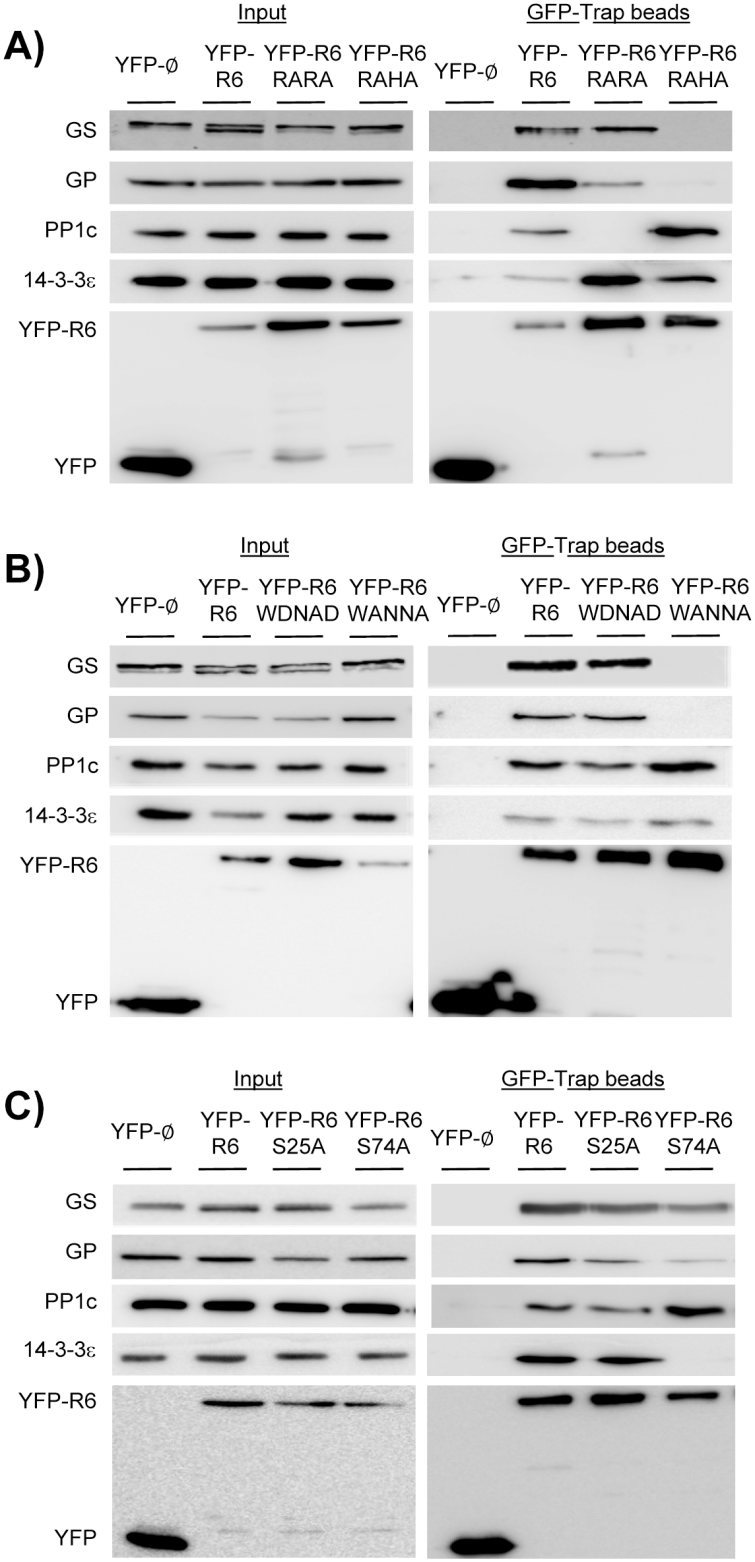
Analysis of the interacting properties of different domains of R6 by immunoprecipitation (GFP-Trap) in mammalian cells. Hek293 cells were transiently transfected with expression vectors coding for YFP, YFP-R6 wild type, and the corresponding mutants YFP-R6 RARA and YFP-R6 RAHA (A), YFP-R6 WDNAD and YFP-R6 WANNA (B), or YFP-R6 S25A and YFP-R6 S74A plasmids (C). Immunoprecipitation analyses were performed using GFP-Trap system (see Materials and Methods section). 40 μL of eluted beads and thirty micrograms of total protein from the soluble fraction of cell lysates (input) were analyzed by SDS-PAGE and Western blotting using appropriated antibodies.

To assess the functionality of the different domains of R6 that we have described above, we generated different mutants in the corresponding protein motifs and checked whether the interaction with different partners was affected. We analyzed first the putative RVXF motifs involved in the interaction with PP1c. The hydrophobic residues valine and phenylalanine within the RVXF motifs were substituted for alanine, resulting in R_102_ARA and R_252_AHA mutants. By yeast two-hybrid analyses we checked the interaction of these mutated forms with the PP1 catalytic subunit (PP1c). In addition, we checked their interaction with laforin, a glucan phosphatase involved in Lafora disease, and with 14-3-3ε proteins, as they are potential interaction partners of R6 [18]. Unfortunately the interaction of R6 with glycogenic substrates, such as glycogen synthase (GS), was undetectable by yeast two-hybrid, what precluded the analysis of the effect of the generated mutations on the interaction with this substrate using this technique. As shown in Fig 2A, mutations in the R_102_VRF motif (R6-RARA mutant) disrupted the binding to PP1c, although the binding to other interacting partners (laforin and 14-3-3ε) remained as wild type. However, mutations in the R_252_VHF motif (R6-RAHA mutant) impaired the binding to PP1c and also to the other interaction partners, despite the construct was successfully produced, suggesting that the mutations had affected the overall structure of the protein when expressed in yeast (Fig 2A). In order to confirm these results, we performed an immunoprecipitation analysis in mammalian Hek293 cells expressing YFP-tagged versions of either R6-RARA or R6-RAHA mutants and compared the results with the wild type form of R6. As shown in Fig 3A, using the GFP-Trap technique, we confirmed that YFP-R6-RARA had completely lost the interaction to endogenous PP1c. This mammalian system allowed us to study the interaction of the different mutants with endogenous glycogenic substrates. In this sense, we observed that the YFP-R6-RARA mutant was still able to interact with endogenous GS and GP (although to at a lesser extend in the latter case), thus indicating that binding to these PP1 substrates was independent to the binding to PP1c. Binding of YFP-R6-RARA to endogenous 14-3-3ε protein was also not affected (Fig 3A). We were not able to check the binding to endogenous laforin as the levels of this protein were very low in these cells (not shown). On the contrary, we found that the YFP-R6-RAHA mutant was able to interact with the endogenous PP1c catalytic subunit (Fig 3A), therefore discarding the R_252_VHF motif as an area of contact to PP1c. The discrepancy between these results and the data obtained by yeast two-hybrid could be due to the different expression systems, yeast *vs* mammalian cells, being the latter more complete as it has endogenous levels of all glycogenic enzymes. Although the R6-RAHA mutant interacted with endogenous 14-3-3ε proteins, it had an impaired interaction with the endogenous PP1 substrates GS and GP (Fig 3A). Probably, the introduced mutations could have altered the conformation of the protein and affected the functionality of the W_267_DNND substrate binding motif present in the near vicinity (Fig 1B) (see below).

### Assessing the functionality of the WDNND substrate binding motif in R6

As indicated above, R6 contains a W_267_DNND motif possibly involved in binding to PP1 substrates (Fig 1A, cyan box). To check the functionality of this region, and since it has been described that in the case of the motif present in R5/PTG the acidic residues (Asp and Glu in the murine form of R5/PTG) were key for substrate binding [11], we decided to substitute the two Asp residues present in the R6 motif (Asp268 and Asp271) to alanine, resulting in the R6-WANNA mutant. Alternatively, we decided to replace the second asparagine residue (Asn270) to alanine, resulting in R6-WDNAD mutant. We expressed these mutated forms in yeast and analyzed their interaction with PP1c, laforin and 14-3-3ε proteins by yeast two-hybrid. We observed that the R6-WDNAD mutant presented a similar interaction pattern as wild type with all the studied proteins: PP1c, laforin and 14-3-3ε proteins (Fig 2B). However, the R6-WANNA mutant did not interact with any of the studied proteins, despite being expressed in yeast (Fig 2B). In order to study the interaction profile of these mutants in a mammalian system, we constructed the corresponding YFP-fusion proteins (YFP-R6-WDNAD and YFP-R6-WANNA) and expressed them in Hek293 cells. As shown in Fig 3B, the R6-WDNAD mutant was able to interact with endogenous PP1c, GS, GP and 14-3-3ε proteins, suggesting that the mutation had not affected the binding properties of R6. On the contrary, the R6-WANNA mutant, although conserved the ability to interact with endogenous PP1c and 14-3-3ε proteins, the binding to the glycogenic substrates GP and GS was severely impaired (Fig 3B). These results confirmed the functionality of the W_267_DNND motif of R6 in substrate binding.

Taking all these results together, we suggest that binding of R6 to PP1c occurs through the R_102_VRF motif and binding of R6 to PP1 substrates occurs in a region comprising the R_252_VHF and the W_267_DNND motifs, being the binding to PP1c and PP1 substrates independent from each other. On the other hand, binding of R6 to 14-3-3ε proteins is independent from these defined regions of R6.

### Defining the 14-3-3 protein binding motif in R6

It is known that 14-3-3 proteins bind to Ser/Thr phosphorylated residues [19]. So, in order to find the putative 14-3-3 binding domain in R6, we searched in the databases for reports on the phosphorylation of R6 and found that it could be potentially phosphorylated in different residues: Ser23, Ser25, Ser28, Ser46, Ser74, Ser77, Ser78 and Ser133 ([32], [33], [34], [35]). However, only two of these sites, Ser25 and Ser74, could form part of the main putative 14-3-3 protein binding consensus motif-RSXpSXP- [19] (Fig 1A, yellow boxes). In order to study the functionality of these sites on 14-3-3 protein binding, we produced non-phosphorylatable mutants in which Ser25 or Ser74 were changed to alanine (S25A, S74A). Then, we assessed the binding properties of the mutated forms by yeast two-hybrid analysis. As shown in Fig 2C, binding of both R6-S25A and R6-S74A to the PP1c catalytic subunit and to laforin was similar to wild type. However, although mutation at Ser25 did not affect the interaction with 14-3-3ε proteins, mutation at Ser74 completely eliminated this interaction (Fig 2C). To confirm these results in a mammalian system, we constructed the corresponding YFP-fusion proteins (YFP-R6-S25A and YFP-R6-S74A), expressed them in Hek293 cells and carried out a GFP-trap analysis. As shown in Fig 3C, R6-S25A mutant interacted with endogenous PP1c, GS, GP and 14-3-3ε as wild type. In the case of the R6-S74A mutant it maintained the interaction with endogenous PP1c, GS and GP (although in this latter case at lower levels), however the interaction with endogenous 14-3-3ε was abolished (Fig 3C). All these results indicated that Ser74, included in the RARS_74_LP motif, plays a crucial role in binding to 14-3-3 proteins, being this interaction independent of the binding of R6 to PP1c and to PP1 glycogenic substrates.

### Role of the different protein binding motifs on the glycogenic activity of R6

**Fig 4:**
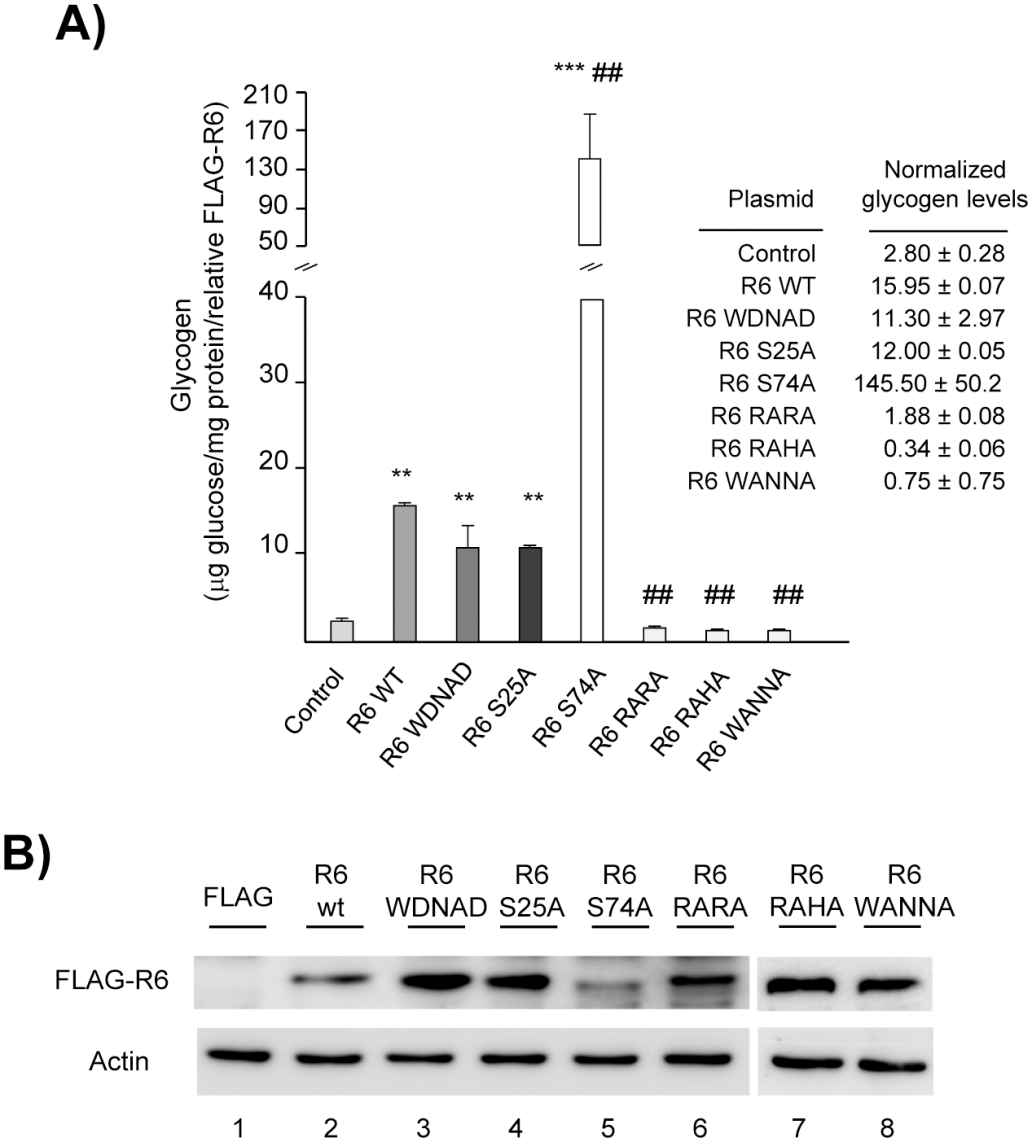
Glycogenic activity of different mutated forms of R6. (A) Measurement of glycogenic activity of different R6 mutated forms. N2a cells were transfected using 1 μg of pFLAG plasmid (negative control), pFLAG-R6 plasmid or its corresponding mutants. Forty-eight hours after transfection, the amount of glycogen was determined as described in Materials and Methods and represented as μg of glucose/mg of protein/relative amount of R6 respect to actin (wild type value considered as 1). Bars indicate standard deviation of three independent experiments (**p<0.01 or ***p<0.001, compared with control cells transfected with an empty plasmid; ##p<0.01, compared with cells expressing R6-WT). An inset with the mean values +/- standard deviation is included. (B) Protein levels of FLAG-R6 forms. A representative western blot analysis is shown. Cell extracts (30 μg) were analyzed using the corresponding anti-FLAG and anti-actin antibodies.

We have previously described that the expression of R6 in a neuroblastoma cell line (N2a) triggers *de novo* glycogen synthesis. In these cells glycogen production is fully dependent on the expression of functional PP1 glycogen targeting subunits since in its absence, glycogen production is very low. The expression of PP1 glycogen targeting subunits, as R6, induces the dephosphorylation of endogenous GS leading to its activation, resulting in glycogen production [17]. To assess the glycogenic activity (capacity to induce glycogen synthesis) of the different R6 mutants we have described above, we expressed FLAG-tagged versions of them in N2a cells and measured the glycogen levels after 48 h of transfection. Consistent with previous results, expression of wild type R6 promoted the accumulation of glycogen (expressed as μg glucose/mg protein/relative amount of FLAG-R6) (Fig 4A). Expression of the R6-RARA mutant, which cannot bind to PP1c but binds to PP1 glycogenic substrates (GS, GP; see above), did not support the promotion of glycogen production (similar levels of glycogen were measured as in cells transfected with an empty plasmid). Next, we analyzed the mutants that affected substrate binding. When R6-RAHA and R6-WANNA mutants were expressed in N2a cells (they do not bind to endogenous GS and GP enzymes; see above), the capacity to support glycogen production was impaired as well, resulting in undetectable levels of glycogen. The expression of the R6-WDNAD mutant, which interacts with PP1c and PP1 glycogenic substrates as wild type (see above), produced amounts of glycogen comparable to the wild type protein. In summary, mutations in R6 affecting the interaction with either PP1c or PP1 glycogenic substrates resulted in an impairment of the glycogenic activity of the mutated forms.

**Fig 5:**
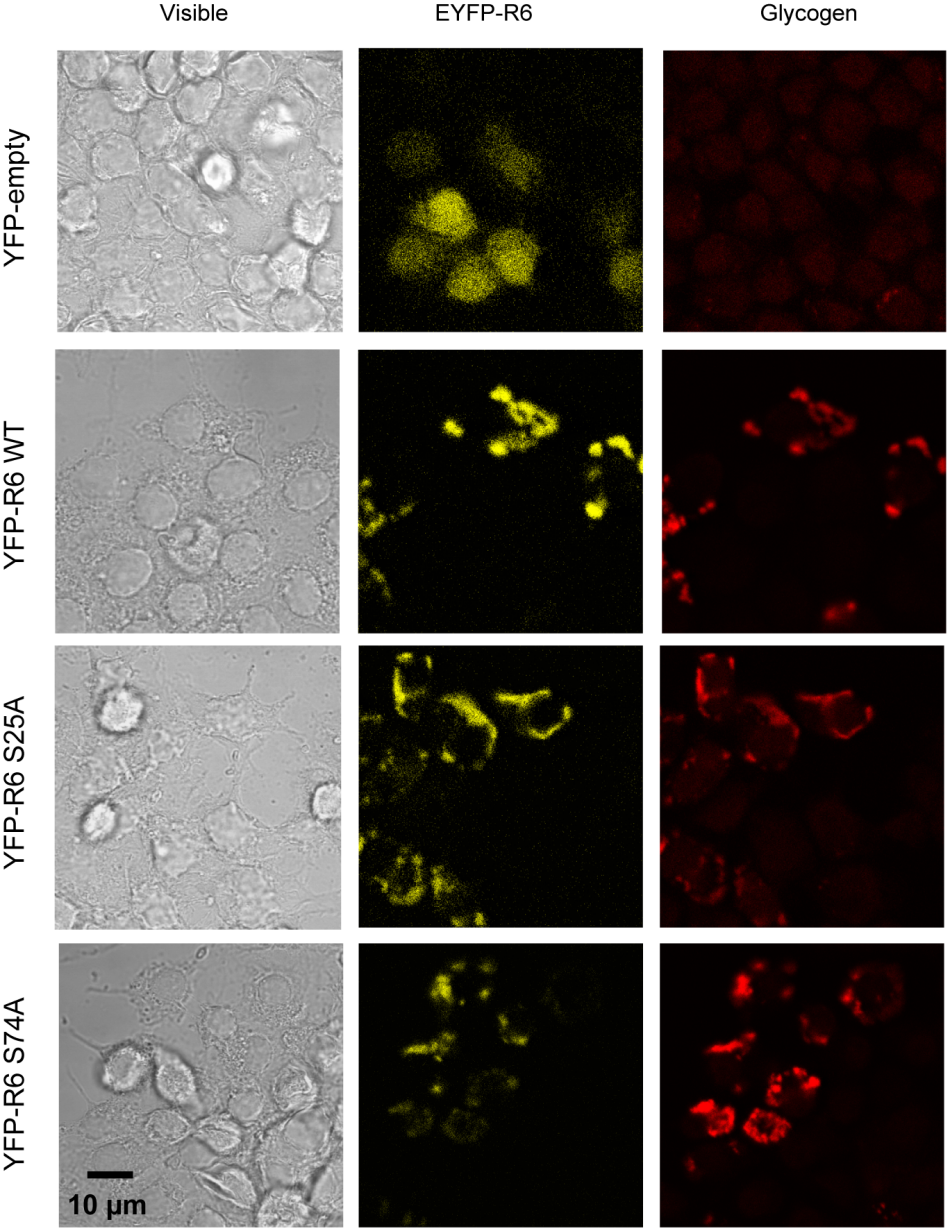
Subcellular localization of R6 S25A and R6 S74A mutated forms. N2a cells were transfected with pYFP empty plasmid, pYFP-R6 wild type, pYFP-R6 S25A or pYFP-R6 S74A plasmids. The subcellular localization of R6 forms and glycogen granules was carried out as described in Materials and Methods. Images were obtained by using confocal microscopy (bars indicate 10 μm). Images corresponding to visible, YFP (in yellow) and glycogen (in red) fluorescences are shown.

We further investigated whether the binding of R6 to 14-3-3 proteins could affect the glycogenic properties of R6. We found that R6-S25A mutant was as glycogenic as wild type (Fig 4A). Surprisingly, the expression of R6-S74A mutant, which is not able to bind to 14-3-3ε proteins (see above), produced around 9 fold increase on glycogen accumulation (Fig 4A). In order to explain the hyper-glycogenic properties of the R6-S74A mutant and since it has been reported that binding to 14-3-3 proteins can affect the subcellular localization of a particular protein [19], we investigated whether the lack of 14-3-3 protein binding present in R6-S74A could have modified the subcellular localization of this protein. So, we expressed in N2a cells the YFP-R6-S25A and YFP-R6-S74A mutants and assessed the subcellular distribution of these proteins. As shown in Fig 5, R6-WT, R6-S25A and R6-S74A located in similar granular structures in the cytoplasm of N2a which contained glycogen, confirming previous results [17]. So, we did not observe any change in the localization of the different mutants in comparison to the wild type protein (Fig 5).

### Binding of 14-3-3 to R6 prevents its lysosomal degradation

**Fig 6:**
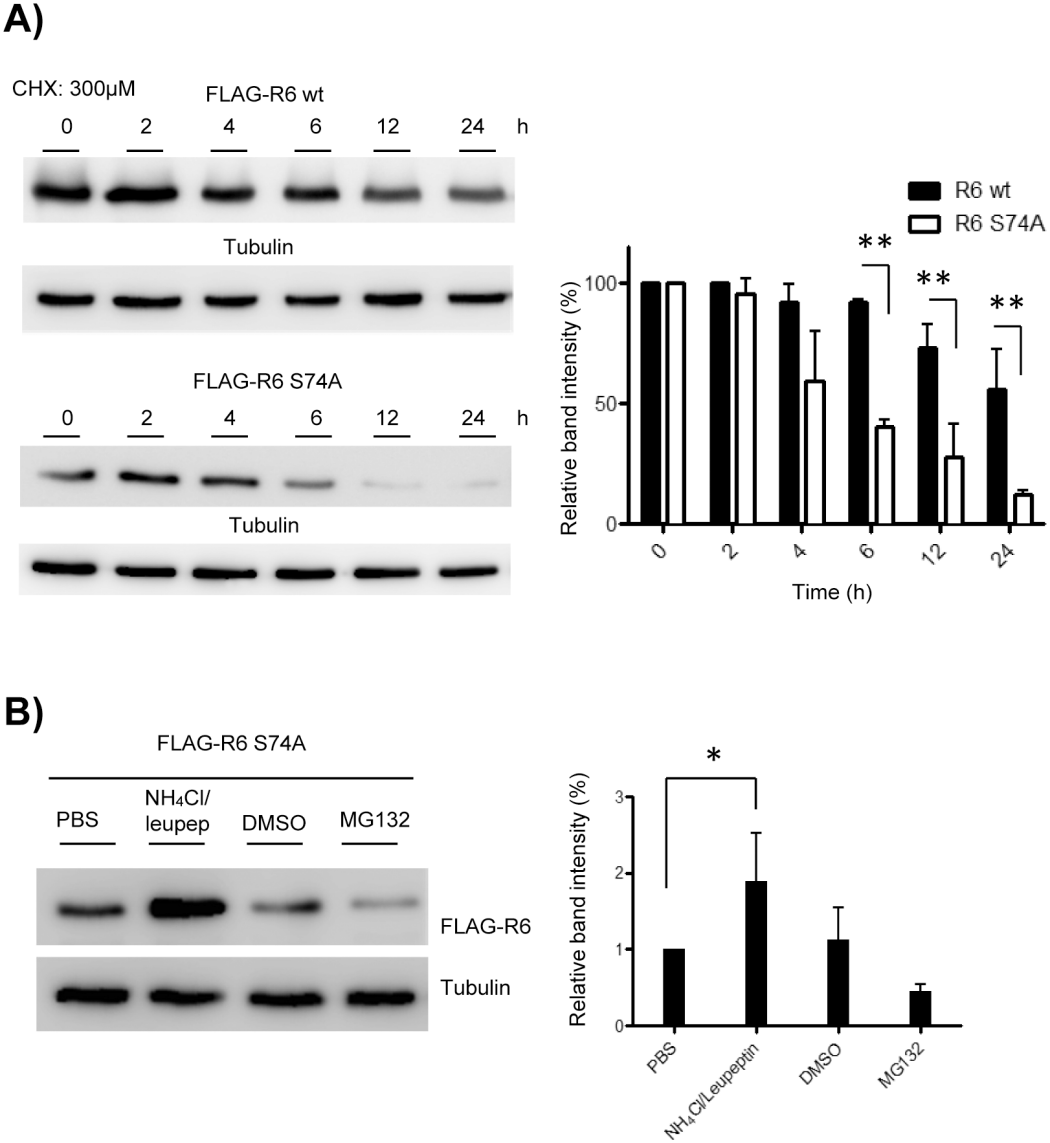
Binding of 14-3-3 proteins to R6 prevents its lysosomal degradation. A) R6-S74A mutant possesses a shorter half-life than wild type protein. Hek293 cells were transfected with pFLAG-R6 wt or pFLAG-R6-S74A plasmids. 24 hours after transfection, cells were treated with cycloheximide (300 μM) to block protein synthesis. At the indicated times, cell extracts (30 μg) were analyzed by Western blotting using anti-FLAG and anti-tubulin (as loading control). A representative western blot is shown on the left panel. On the right panel, the intensity of the bands related to the levels of tubulin is plotted and normalized respect to the values at time 0 (bars indicate the standard deviation of at least three independent experiments; **p < 0.01). B) R6-S74A protein is degraded by the lysosomal pathway. Hek293 cells were transfected with pFLAG-R6-S74A plasmid. Eighteen hours after transfection, cells were treated with ammonium chloride (20 mM)/ leupeptin (100 μM) or MG132 (5 μM) for six hours. Then, cells were lysed and extracts (30 μg) were analyzed by immunoblotting using anti-FLAG antibody and anti-tubulin as loading control. The intensity of the bands related to the levels of tubulin is plotted (bars indicate standard deviation of at least three independent experiments; *p < 0.05).

In the course of the subcellular localization experiments described above, we noticed that the YFP-R6-S74A protein was expressed at much lower levels than the wild type or the R6-S25A mutant (Fig 5). Similarly, lower levels of FLAG-R6-S74A were observed in Fig 4B (lane 5). In order to analyze if mutation at Ser74 was affecting R6 stability, we performed an assay to compare the half-life of this mutated form to the wild type protein. We expressed in Hek293 cells either the FLAG-R6 wild type or the FLAG-R6-S74A mutant and treated the cells with cycloheximide to block *de novo* protein synthesis. Then, protein levels were measured by western blotting at different times after the treatment. As observed in Fig 6A, the R6-S74A protein had a shorter half-life than the wild type protein. After 24h of treatment, the R6-S74A mutant was degraded almost completely in comparison to the wild type form, which was rather stable (Fig 6A). To elucidate which mechanism of degradation was taking place, we treated the cells with either MG132, to inhibit proteasome function, or with leupeptin and NH_4_Cl to inhibit lysosomal degradation [36]. We observed that treatment with MG132 did not affect the degradation of R6-S74A protein (Fig 6B). On the contrary, treatment with leupeptin and NH_4_Cl (to block the lysosome) prevented the degradation of the R6-S74A mutated form (Fig 6B). Therefore, disrupting the binding of 14-3-3 proteins to R6 accelerated its degradation by the lysosomal pathway.

## Discussion

**Fig 7:**
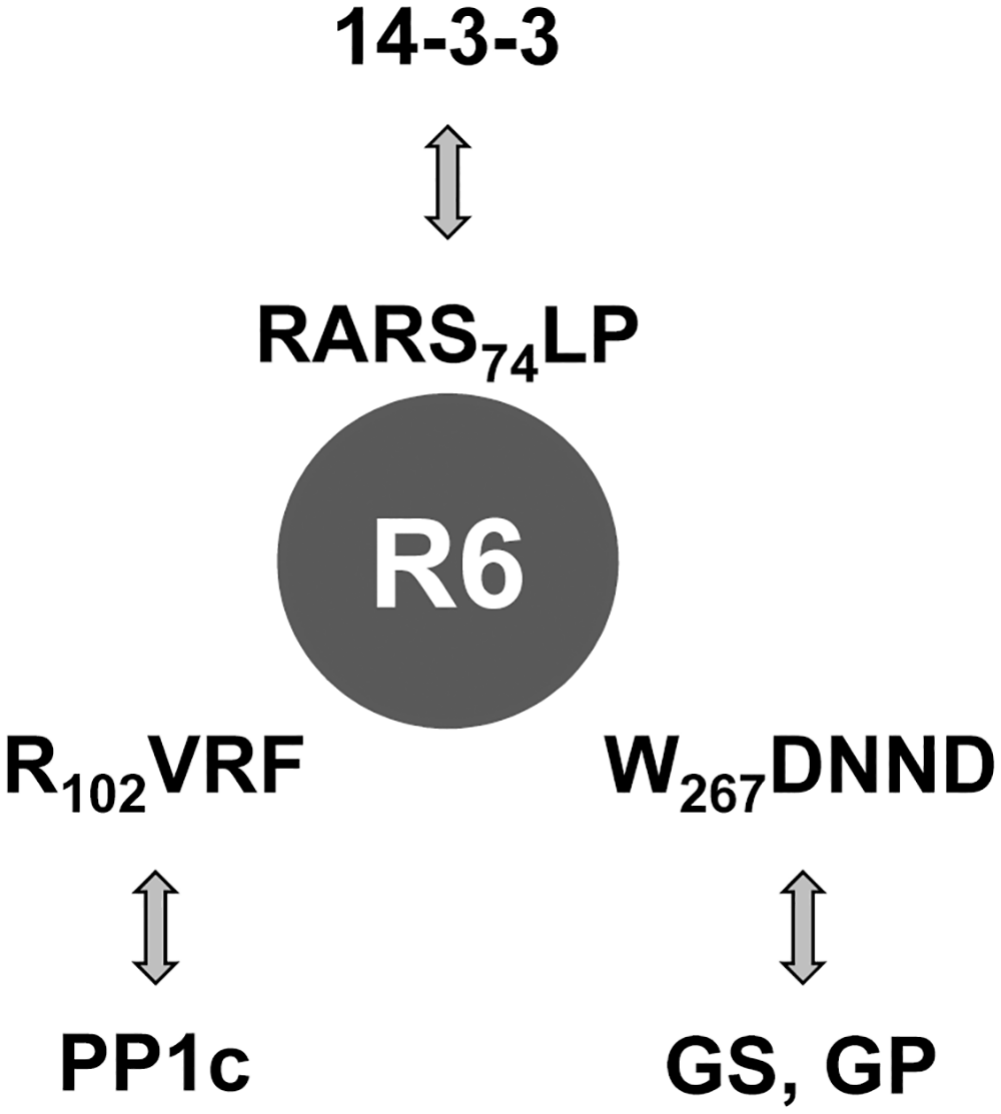
Schematic representation of the different binding regions in R6. R6 possesses three separated interaction domains: PP1c binding motif (R_102_VRF), PP1 substrate binding region (W_267_DNND) and 14-3-3 binding motif (RARS_74_LP). GS, glycogen synthase; GP, glycogen phosphorylase.

Protein phosphatase 1 (PP1) plays a crucial role in regulating glycogen synthesis. It dephosphorylates key enzymes involved in glycogen homeostasis, such as glycogen synthase (GS) and glycogen phosphorylase (GP), leading to the activation of the former and the inactivation of the latter, resulting in glycogen accumulation. However, PP1 does not interact directly with GS or GP but binds to specific regulatory subunits that target PP1 to the glycogenic substrates. To perform their function these PP1 glycogen targeting subunits must bind, on one hand to PP1 catalytic subunit (PP1c) and on the other hand to PP1 glycogenic substrates ([1], [3]). In this work we have carried out a structure-function analysis of the different protein binding domains we have identified in one of these glycogen targeting subunits, namely R6 (PPP1R3D) (Fig 7). Our data indicates that R6 contains a typical RVXF motif (R_102_VRF) involved in PP1c binding (Fig 7). This motif is also present in the other major glycogen targeting subunits studied so far [PPP1R3A (GM): residues R_63_VSF; PPP1R3B (GL): residues R_62_VSF; PPP1R3C (R5/PTG): R_84_VVF; numbering refers to the human counterpart of each protein, according to Uniprot]. We demonstrate that in R6 this motif is essential to maintain the ability of the protein to induce glycogen synthesis. We also present data indicating that R6 contains a region (W_267_DNND) involved in binding to glycogenic substrates (GS and GP) (Fig 7). This motif is well conserved in other glycogen targeting subunits [PPP1R3A (GM): residues W_219_SNNN; PPP1R3B (GL): residues W_222_DSNR; PPP1R3C (R5/PTG): residues W_246_DNND] but, to our knowledge, only in the case of R6 (this work) and in the case of murine R5/PTG [11] and rabbit GM [10], the functionality of this motif in glycogen synthesis has been evaluated. Consistent with its binding properties, this domain is well exposed to the solvent in both the model we present for R6 (Fig 1B) and in the case of GL (pdb accession code: 2EEF). Our work indicates that R6 contains an additional region regulating binding to PP1 glycogenic substrates, which is essential for the glycogenic activity of the targeting subunit: R_256_VHF (Fig 7). Although not conserved, a similar region is present in R5/PTG (K_231_IEF) and GL (R_207_MEF) (Fig 1A), raising the possibility that it could form part of an extended area of contact with PP1 glycogenic substrates that would comprise in R6 from R_256_VHF to the W_267_DNND region (Fig 1B). Alternatively, and since the hydrophobic residues (Val and Phe) in the R_256_VHF domain (mutated in the R6-RAHA form which has impaired interaction with glycogenic substrates) are buried and not exposed to the solvent in the structural model we present (Fig 1B) and also in the case of GL (pdb accession code: 2EEF), they could participate in intramolecular contacts, so when they are changed to Ala residues a conformational modification in this area could happen, affecting the binding properties of the W_267_DNND motif that is nearby (Fig 1B). In any case, and in agreement with a previous report [11], our results indicate that binding of R6 to PP1c and PP1 glycogenic substrates are independent processes, although impairment of any of them results in the same loss of functionality in glycogen homeostasis.

We also report a novel functional domain in R6 involved in binding to 14-3-3 proteins (RARS_74_LP) (Fig 7). This domain is absent in other glycogen targeting subunits as GL or R5/PTG (Fig 1A); in fact, we were not able to detect any interaction of these glycogenic subunits with 14-3-3 proteins by yeast two-hybrid (not shown). Mutation in the critical Ser74 residue of R6 disrupts binding to 14-3-3ε proteins, although it still allows the binding to PP1c and to PP1 glycogenic substrates. As different mass spectrometry analyses indicate that Ser74 is phosphorylated *in vivo* ([32], [33], [34], [35]), binding of R6 to 14-3-3 proteins could protect this site from dephosphorylation and prevent the rapid degradation of the protein by the lysosomal pathway. It is surprising that a form that cannot be phosphorylated at Ser74 (and hence it cannot bind 14-3-3 proteins) presented hyper-glycogenic properties. In our hands, the expression of even very low amounts of the R6-S74A protein induced the production of high levels of glycogen. Perhaps this R6-S74A protein has better dephosphorylation kinetics against glycogenic enzymes or, alternatively, it could affect other unknown regulators of glycogen synthesis. The fact that the R6-S74A form is very unstable may define protein degradation as a novel pathway in the control of the glycogenic properties of R6.

In conclusion, in this work we present strong evidence demonstrating the functionality of different protein domains in R6: the PP1c-binding motif, the PP1 substrate motif and the 14-3-3 protein binding motif. All of them play their independent role in providing a docking region for interaction with their corresponding partners.

## References

[1] Roach PJ, Depaoli-Roach AA, Hurley TD, Tagliabracci VS (2012) Glycogen and its metabolism: some new developments and old themes. Biochem J 441: 763–787. 10.1042/BJ20111416 22248338 PMC4945249

[2] Bollen M, Peti W, Ragusa MJ, Beullens M (2010) The extended PP1 toolkit: designed to create specificity. Trends Biochem Sci 35: 450–458. 10.1016/j.tibs.2010.03.002 20399103 PMC3131691

[3] Heroes E, Lesage B, Gornemann J, Beullens M, Van Meervelt L, Bollen M (2013) The PP1 binding code: a molecular-lego strategy that governs specificity. FEBS J 280: 584–595. 10.1111/j.1742-4658.2012.08547.x 22360570

[4] Hendrickx A, Beullens M, Ceulemans H, Den Abt T, Van Eynde A, Nicolaescu E, et al (2009) Docking motif-guided mapping of the interactome of protein phosphatase-1. Chem Biol 16: 365–371. 10.1016/j.chembiol.2009.02.012 19389623

[5] Munro S, Ceulemans H, Bollen M, Diplexcito J, Cohen PT (2005) A novel glycogen-targeting subunit of protein phosphatase 1 that is regulated by insulin and shows differential tissue distribution in humans and rodents. Febs J 272: 1478–1489. 15752363 10.1111/j.1742-4658.2005.04585.x

[6] Aggen JB, Nairn AC, Chamberlin R (2000) Regulation of protein phosphatase-1. Chem Biol 7: R13–23. 10662690 10.1016/s1074-5521(00)00069-7

[7] Newgard CB, Brady MJ, O'Doherty RM, Saltiel AR (2000) Organizing glucose disposal: emerging roles of the glycogen targeting subunits of protein phosphatase-1. Diabetes 49: 1967–1977. 11117996 10.2337/diabetes.49.12.1967

[8] Printen JA, Brady MJ, Saltiel AR (1997) PTG, a protein phosphatase 1-binding protein with a role in glycogen metabolism. Science 275: 1475–1478. 9045612 10.1126/science.275.5305.1475

[9] Kelsall IR, Voss M, Munro S, Cuthbertson DJ, Cohen PT (2011) R3F, a novel membrane-associated glycogen targeting subunit of protein phosphatase 1 regulates glycogen synthase in astrocytoma cells in response to glucose and extracellular signals. J Neurochem 118: 596–610. 10.1111/j.1471-4159.2011.07345.x 21668450

[10] Liu J, Brautigan DL (2000) Glycogen synthase association with the striated muscle glycogen-targeting subunit of protein phosphatase-1. Synthase activation involves scaffolding regulated by beta-adrenergic signaling. J Biol Chem 275: 26074–26081. 10856301 10.1074/jbc.M003843200

[11] Fong NM, Jensen TC, Shah AS, Parekh NN, Saltiel AR, Brady MJ (2000) Identification of binding sites on protein targeting to glycogen for enzymes of glycogen metabolism. J Biol Chem 275: 35034–35039. 10938087 10.1074/jbc.M005541200

[12] Machovic M, Janecek S (2006) Starch-binding domains in the post-genome era. Cell Mol Life Sci 63: 2710–2724. 17013558 10.1007/s00018-006-6246-9 PMC11135984

[13] Christiansen C, Abou Hachem M, Janecek S, Vikso-Nielsen A, Blennow A, Svensson B (2009) The carbohydrate-binding module family 20—diversity, structure, and function. FEBS J 276: 5006–5029. 10.1111/j.1742-4658.2009.07221.x 19682075

[14] Armstrong CG, Browne GJ, Cohen P, Cohen PT (1997) PPP1R6, a novel member of the family of glycogen-targetting subunits of protein phosphatase 1. FEBS Lett 418: 210–214. 9414128 10.1016/s0014-5793(97)01385-9

[15] Esteves SL, Domingues SC, da Cruz e Silva OA, Fardilha M, da Cruz e Silva EF (2012) Protein phosphatase 1alpha interacting proteins in the human brain. OMICS 16: 3–17. 10.1089/omi.2011.0041 22321011 PMC3275796

[16] Montori-Grau M, Guitart M, Garcia-Martinez C, Orozco A, Gomez-Foix AM (2011) Differential pattern of glycogen accumulation after protein phosphatase 1 glycogen-targeting subunit PPP1R6 overexpression, compared to PPP1R3C and PPP1R3A, in skeletal muscle cells. BMC Biochem 12: 57 10.1186/1471-2091-12-57 22054094 PMC3240831

[17] Rubio-Villena C, Garcia-Gimeno MA, Sanz P (2013) Glycogenic activity of R6, a protein phosphatase 1 regulatory subunit, is modulated by the laforin-malin complex. Int J Biochem Cell Biol 45: 1479–1488. 10.1016/j.biocel.2013.04.019 23624058

[18] Benzinger A, Muster N, Koch HB, Yates JR 3rd, Hermeking H (2005) Targeted proteomic analysis of 14-3-3 sigma, a p53 effector commonly silenced in cancer. Mol Cell Proteomics 4: 785–795. 15778465 10.1074/mcp.M500021-MCP200

[19] Obsil T, Obsilova V (2011) Structural basis of 14-3-3 protein functions. Semin Cell Dev Biol 22: 663–672. 10.1016/j.semcdb.2011.09.001 21920446

[20] Parua PK, Young ET (2014) Binding and transcriptional regulation by 14-3-3 (Bmh) proteins requires residues outside of the canonical motif. Eukaryot Cell 13: 21–30. 10.1128/EC.00240-13 24142105 PMC3910956

[21] Fernandez-Sanchez ME, Criado-Garcia O, Heath KE, Garcia-Fojeda B, Medrano-Fernandez I, Gomez-Garre P, et al (2003) Laforin, the dual-phosphatase responsible for Lafora disease, interacts with R5 (PTG), a regulatory subunit of protein phosphatase-1 that enhances glycogen accumulation. Hum Mol Genet 12: 3161–3171. 14532330 10.1093/hmg/ddg340

[22] Garcia-Haro L, Garcia-Gimeno MA, Neumann D, Beullens M, Bollen M, Sanz P (2010) The PP1-R6 protein phosphatase holoenzyme is involved in the glucose-induced dephosphorylation and inactivation of AMP-activated protein kinase, a key regulator of insulin secretion, in MIN6 beta cells. Faseb J 24: 5080–5091. 10.1096/fj.10-166306 20724523

[23] Lesage B, Beullens M, Pedelini L, Garcia-Gimeno MA, Waelkens E, Sanz P, et al (2007) A complex of catalytically inactive protein phosphatase-1 sandwiched between Sds22 and inhibitor-3. Biochemistry 46: 8909–8919. 17630778 10.1021/bi7003119

[24] Paumi CM, Menendez J, Arnoldo A, Engels K, Iyer KR, Thaminy S, et al (2007) Mapping protein-protein interactions for the yeast ABC transporter Ycf1p by integrated split-ubiquitin membrane yeast two-hybrid analysis. Mol Cell 26: 15–25. 17434123 10.1016/j.molcel.2007.03.011

[25] Ludin K, Jiang R, Carlson M (1998) Glucose-regulated interaction of a regulatory subunit of protein phosphatase 1 with the Snf1 protein kinase in Saccharomyces cerevisiae. Proc Natl Acad Sci U S A 95: 6245–6250. 9600950 10.1073/pnas.95.11.6245 PMC27646

[26] Combet C, Blanchet C, Geourjon C, Deleage G (2000) NPS@: network protein sequence analysis. Trends Biochem Sci 25: 147–150. 10694887 10.1016/s0968-0004(99)01540-6

[27] Schwede T, Kopp J, Guex N, Peitsch MC (2003) SWISS-MODEL: An automated protein homology-modeling server. Nucleic Acids Res 31: 3381–3385. 12824332 10.1093/nar/gkg520 PMC168927

[28] Arnold K, Bordoli L, Kopp J, Schwede T (2006) The SWISS-MODEL workspace: a web-based environment for protein structure homology modelling. Bioinformatics 22: 195–201. 16301204 10.1093/bioinformatics/bti770

[29] Benkert P, Kunzli M, Schwede T (2009) QMEAN server for protein model quality estimation. Nucleic Acids Res 37: W510–514. 10.1093/nar/gkp322 19429685 PMC2703985

[30] Lovell SC, Davis IW, Arendall WB 3rd, de Bakker PI, Word JM, Prisant MG, et al (2003) Structure validation by Calpha geometry: phi,psi and Cbeta deviation. Proteins 50: 437–450. 12557186 10.1002/prot.10286

[31] Chan TM, Exton JH (1976) A rapid method for the determination of glycogen content and radioactivity in small quantities of tissue or isolated hepatocytes. Anal Biochem 71: 96–105. 1275237 10.1016/0003-2697(76)90014-2

[32] Dephoure N, Zhou C, Villen J, Beausoleil SA, Bakalarski CE, Elledge SJ, et al (2008) A quantitative atlas of mitotic phosphorylation. Proc Natl Acad Sci U S A 105: 10762–10767. 10.1073/pnas.0805139105 18669648 PMC2504835

[33] Raijmakers R, Kraiczek K, de Jong AP, Mohammed S, Heck AJ (2010) Exploring the human leukocyte phosphoproteome using a microfluidic reversed-phase-TiO2-reversed-phase high-performance liquid chromatography phosphochip coupled to a quadrupole time-of-flight mass spectrometer. Anal Chem 82: 824–832. 10.1021/ac901764g 20058876

[34] Zhou H, Di Palma S, Preisinger C, Peng M, Polat AN, Heck AJ, et al (2013) Toward a comprehensive characterization of a human cancer cell phosphoproteome. J Proteome Res 12: 260–271. 10.1021/pr300630k 23186163

[35] Sharma K, D'Souza RC, Tyanova S, Schaab C, Wisniewski JR, Cox J, et al (2014) Ultradeep human phosphoproteome reveals a distinct regulatory nature of Tyr and Ser/Thr-based signaling. Cell Rep 8: 1583–1594. 10.1016/j.celrep.2014.07.036 25159151

[36] Knecht E, Aguado C, Carcel J, Esteban I, Esteve JM, Ghislat G, et al (2009) Intracellular protein degradation in mammalian cells: recent developments. Cell Mol Life Sci 66: 2427–2443. 10.1007/s00018-009-0030-6 19399586 PMC11115841

